# Three transmission events of *Vibrio cholerae* O1 into Lusaka, Zambia

**DOI:** 10.1186/s12879-021-06259-5

**Published:** 2021-06-14

**Authors:** John Mwaba, Amanda K. Debes, Kelsey N. Murt, Patrick Shea, Michelo Simuyandi, Natasha Laban, Katayi Kazimbaya, Caroline Chisenga, Shan Li, Mathieu Almeida, Jacquelyn S. Meisel, Aaron Shibemba, Timothy Kantenga, Victor Mukonka, Geoffrey Kwenda, David A. Sack, Roma Chilengi, O. Colin Stine

**Affiliations:** 1 Centre for Infectious Disease Research in Zambia, Lusaka, Zambia; 2 Department of Biomedical Sciences, University of Zambia School of Health Sciences, Lusaka, Zambia; 3 Department of Pathology and Microbiology, University Teaching Hospitals, Lusaka, Zambia; 4 Johns Hopkins Bloomberg School of Public Health, MD Baltimore, USA; 5 University of Maryland School of Medicine, Baltimore, MD USA; 6 University of Maryland, College Park, College Park, MD USA; 7 Université Paris-Saclay, INRAE, MGP, 78350 Jouy-en-Josas, France; 8 Zambia National Public Health Institute, Lusaka, Zambia

**Keywords:** Molecular characterization, *V. cholerae*, MLVA, Whole genome sequencing, Zambia

## Abstract

**Background:**

Cholera has been present and recurring in Zambia since 1977. However, there is a paucity of data on genetic relatedness and diversity of the *Vibrio cholerae* isolates responsible for these outbreaks. Understanding whether the outbreaks are seeded from existing local isolates or if the outbreaks represent separate transmission events can inform public health decisions.

**Results:**

Seventy-two *V. cholerae* isolates from outbreaks in 2009/2010, 2016, and 2017/2018 in Zambia were characterized using multilocus variable number tandem repeat analysis (MLVA) and whole genome sequencing (WGS). The isolates had eight distinct MLVA genotypes that clustered into three MLVA clonal complexes (CCs). Each CC contained isolates from only one outbreak. The results from WGS revealed both clustered and dispersed single nucleotide variants. The genetic relatedness of isolates based on WGS was consistent with the MLVA, each CC was a distinct genetic lineage and had nearest neighbors from other East African countries. In Lusaka, isolates from the same outbreak were more closely related to themselves and isolates from other countries than to isolates from other outbreaks in other years.

**Conclusions:**

Our observations are consistent with i) the presence of random mutation and alternative mechanisms of nucleotide variation, and ii) three separate transmission events of *V. cholerae* into Lusaka, Zambia. We suggest that locally, case-area targeted invention strategies and regionally, well-coordinated plans be in place to effectively control future cholera outbreaks.

**Supplementary Information:**

The online version contains supplementary material available at 10.1186/s12879-021-06259-5.

## Background

Cholera has been present and recurring in Zambia since 1977 [1]. In Zambia and in most sub-Saharan countries, cholera is diagnosed using stool culture, serology and rapid diagnostic tests (RDTs) [2]. However, culture and RDTs do not provide the genetic relatedness or diversity information that is critical to having insight into whether the outbreaks are seeded from existing local isolates or if the outbreaks represent separate transmission events, hence, the need to have genetic data in addition to culture and RDT data. If the isolates from one outbreak to the next are genetically closely related then, the outbreaks may have been seeded from existing local isolates. However, if the isolates from successive outbreaks are genetically distinct, then the outbreaks represent separate transmission events of *V. cholerae* into the region. Therefore, to determine the spread of specific genetic lineages of *V*. *cholerae* responsible for cholera outbreaks, the use of advanced molecular testing methods is recommended [3]. Knowledge of genetic differences among isolates facilitates a greater understanding of the transmission within and between geographic regions and time periods [4]. We performed multilocus variable number tandem repeat analysis (MLVA) and whole genome sequencing (WGS) on isolates from cholera outbreaks in 2009/2010, 2016and 2017/2018 in Zambia.

## Methods

This was a cross-sectional study analyzing isolates collected from each region of Zambia where microbiological culture facilities and long-term storage capabilities were available. It was a convenience sample. The isolates were recovered from stools that were plated and grown overnight on thiosulfate-citrate-bile salts-sucrose agar (TCBS). Permission to work on stored isolates was granted by the Ministry of Health and approved by the ethical review committee of The University of Zambia Biomedical Research Ethics Committee (UNZABREC- REF NO:003–10-17). There were no human participants involved. All methods were performed in accordance with the relevant guidelines and regulations. The isolates were preserved on Whatman™ 903 filter paper (GE Healthcare Ltd., Cardiff UK) according to previously published methods and were shipped to Baltimore, Maryland, USA, for molecular testing [5].

DNA extraction and polymerase chain reaction (PCR) confirmation of the isolates was done according to previously described methods [6]. PCR products of *ompW (*outer membrane protein*)* and*ctxA (cholera enterotoxin sub-unit A)* were separated by electrophoresis through 1.5% agarose gels, stained with ethidium bromide and visualized with UV light.

Upon confirmation, DNA from *V. cholerae* O1 isolates was amplified by PCR at each of five previously identified variable number of tandem repeat loci (VC0147, VC0437, VC1650, VCA0171 and VCA0283) using fluorescently labeled primers [7]. The labeled fragments were separated using a 3730xl ABI Automatic Sequencer and sized using internal lane standards (LIZ600; ABI, Foster City, CA) with the Gene Mapper v4.0 program (ABI). Alleles were identified by the number of repeats at the locus. EBURST (http://eburst.mlst.net) was used to define the genetic relatedness between genotypes. Genotypes within a clonal complex (CC) were related by a series of single or double locus variants [4]. **The MLVA datasets are available from the corresponding author on request.**

WGS was performed on representatives from each outbreak, year and MLVA CC. Libraries for Illumina sequencing were prepared, enriched, barcoded in ten cycles of PCR amplification with primers containing an index sequence and sequenced using 150 basepaired-end runs on either an Illumina HiSeq2500 or Novaseq6000 (Illumina, San Diego, CA). The quality of the 150 base paired-end reads was assessed with FastQC v0.11.7 [8] and reads were quality trimmed using Fastp (v 0.21.0 with the following flags: --detect_adapter_for_pe --trim_poly_g --thread 12 --average_qual 20 -f 15) [9] and remaining contaminant sequences coming from the phiX genome (NC_001422.1) were filtered out using bowtie2 v2.3.0 [10], samtools v1.5 [11] and bedtools v2.26.0 [12]. High quality reads were assembled with Spades software v.3.14.0 with the --isolate option [13] and the assembled genomes were checked using QUAST v5.0.0.dev0, b385864 [14]. All variants detected within one kb of the end of the contigs were removed. Information about each genome is in Supplemental Table 1. The assembled sequences were submitted to Genbank (https://www.ncbi.nlm.nih.gov/) accession number PRJNA701309. Parsnp (v1.2) was used to align the variable nucleotides to generate variant and alignment description files. Gingr (v1.2) was used to visualize the alignment file and export a multiple alignment file [15]. To understand the genetic relatedness of the Zambian strains, 99 African isolates with known WGS from transmission events T10, T11 and T13 were selected (Supplemental Table 1), analyzed in FastTree2 (v2.1.9) [16] and the maximum likelihood tree was visualized using Interactive Tree of Life (iTOL) [17]. Clustering of variant nucleotides was performed manually using the vcf file from Parsnp (v1.2) [15]. To determine whether the variant nucleotide was in a gene, a 20 nt region surrounding the SNV was submitted to blastn to identify the 59_Luasaka_2010 associated protein and compared by BLAST [18] to the *V. cholerae* MS6 proteins (https://www.ncbi.nlm.nih.gov/genome/?term=cholerae+MS6), which were functionally annotated previously by NCBI. **The scripts for the analyses are available from the corresponding author on reasonable request.**

## Results

**Fig. 1:**
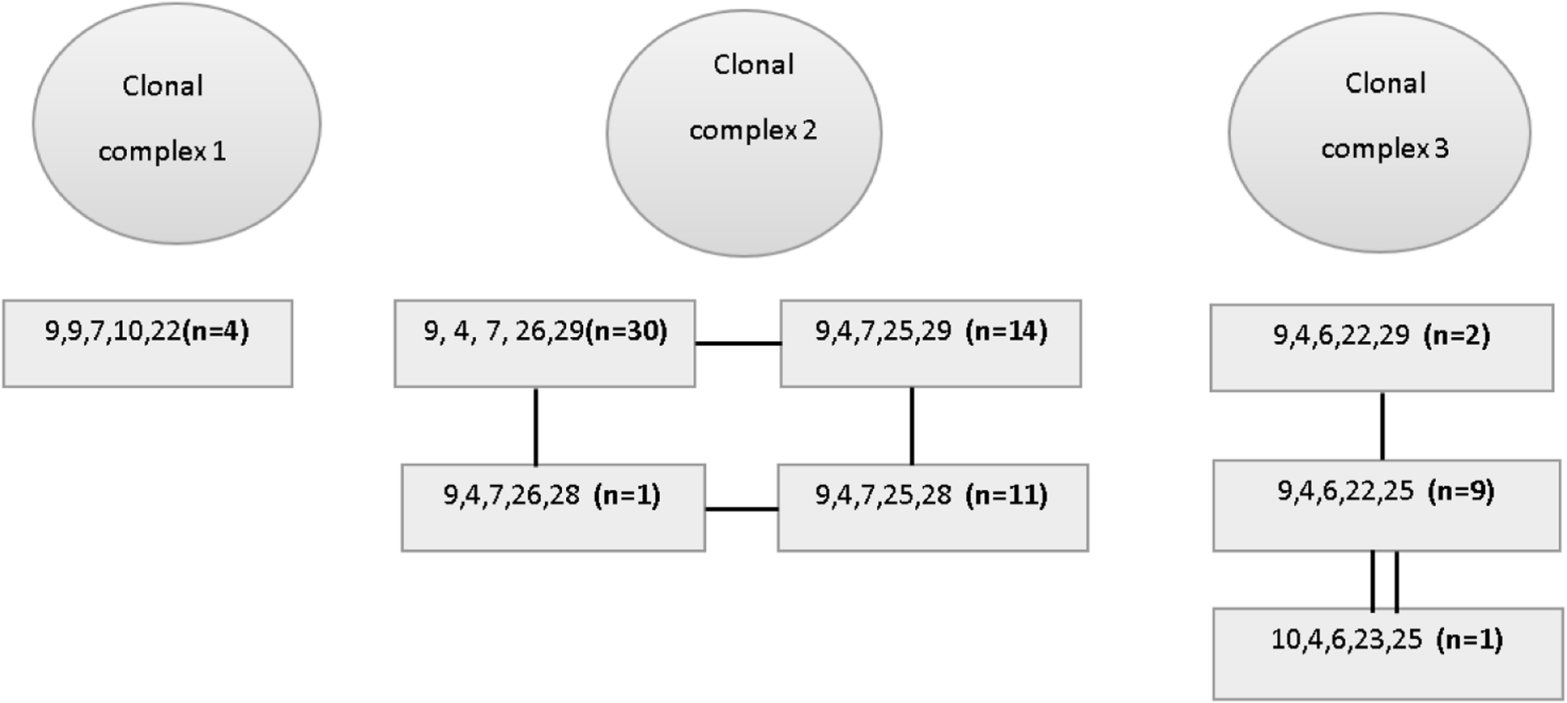
MLVA *V. cholerae* CCs responsible 2009/10, 2016 and 2017/18 cholera outbreaks in Zambia. Each genotype is represented by five numbers corresponding to the number of repeats at the five loci. The lines between the genotypes represents an allelic change at a single locus. n = represents number of isolates with that genotype. Clonal complex 1 (CC1) = Lusaka outbreak (2009/10), CC2 = Lusaka outbreak (2016), CC3 = Chiengi, Mpulungu & Lusaka outbreak (2017/18)

All 72 isolates tested positive for *ompW* and *ctxA*. MLVA was successfully performed on all 72 cholera isolates, revealing eight different genotypes that constituted three CCs responsible for cholera outbreaks in 2009/10, 2016 and 2017/18 in Zambia. CC1 isolates were isolated in Lusaka during the 2009/10 outbreak, CC2 were isolates from Lusaka’s 2016 cholera outbreak, and CC3 is comprised of isolates from Chiengi, Mpulungu and Lusaka during the 2017/2018 outbreak (Fig. 1).

The isolates contained in CC3 had the largest geographical and temporal distribution. An isolate was detected in the northeast district of Mpulungu in 2017. Isolates were also detected in Lusaka (central Zambia) in October 2017 and February 2018. The genotype of the Mpulungu isolate is identical to the most frequent genotype in Lusaka. Although the year of isolation is known, the month is not, thus the direction of the spread cannot be determined. Nevertheless, these observations are consistent with the CC3 genotype spreading between Mpulungu and Lusaka.

**Fig. 2:**
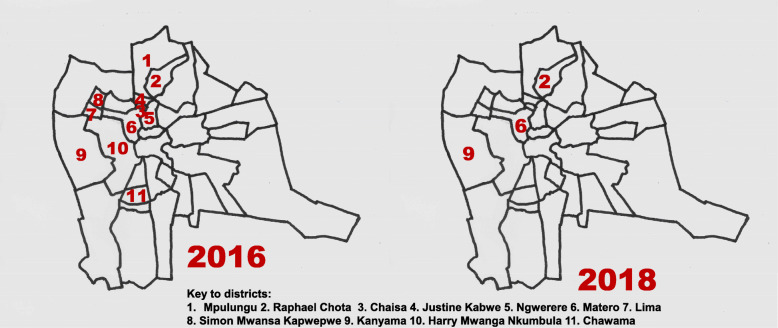
Distribution of cholera in Lusaka by ward 2016 (left) and 2017/2018 (right). Shapefile map available at https://www.citypopulation.de/en/zambia/wards/admin/0504__lusaka

Examining the cholera outbreaks that occurred within the Lusaka district showed that the isolates for the 2016 outbreak came from 11 wards (Mpulungu, Raphael Chota, Chaisa, Justine Kabwe, Ngwerere, Matero, Lima, Simon Mwansa Kapwepwe, Kanyama, Harry Mwanga Nkumbula, Chawama), while those collected during the 2017/18 outbreak came from 3 wards (Raphael Chota, Matero, Kanyama) (Fig. 2). Significantly, the genotypes of 2016 isolates comprised CC2, while the 2017/2018 isolates comprised CC3.

**Fig. 3:**
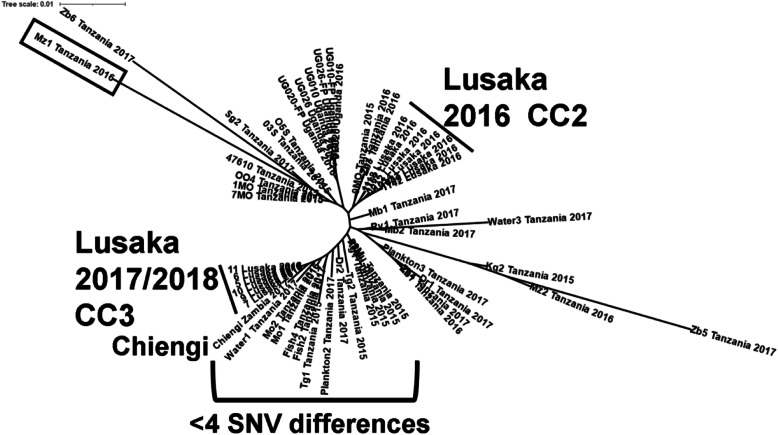
Unrooted phylogram of WGS from T13 *V. cholerae* isolates from Zambia, Tanzania and Uganda. The branch lines are proportional to the number of nucleotide differences between the sequences. CC2 and CC3 refer to the clonal complex defined by MLVA. The box indicates isolate Mz1 from Tanzania collected in 2016 that is an example of an isolate with many unique SNVs as seen by the long branch length. The seven isolates collected in Lusaka in 2016 are on a distinct branch from the six isolates collected 2017/2018 in Lusaka. The 2017/2018 isolates from Lusaka are less than 4 SNV differences from thirteen isolates from Tanzania collected in 2015, 2016 and 2017. The Chiengi isolate is 4 unique SNVs from the 2017/2018 isolates from Lusaka

Whole genome sequencing of 18 isolates revealed that all were from wave three of the seventh pandemic. Those isolated in 2009 and 2010 belonged to the tenth transmission event (T10) from south Asia to Africa, while those from 2016, and 2017/2018 were from T13 (Supplemental Table 1). The isolates from Zambia within an outbreak are all very closely related to each other and further away from the other Zambia isolates from other outbreaks in other years than from isolates from neighboring countries (Supplemental Figure 1). The T13 isolates from Lusaka were all closely related forming two clusters: one containing the 2016 isolates and the second containing those from 2017/2018 (Fig. 3 and Supplemental Figure 1). Remarkably, the 2016 isolates were three single nucleotide variants (SNVs) or less different from each other and four or more SNVs different from the 2017/2018 isolates that did not differ among themselves (Supplemental Table 2). The distinctness between the 2016 and 2017/2018 isolates is supported by two observations. The first was when DNA was re-extracted and sequenced from the same isolate, the pair of genomes differed by 0 to 2 SNVs (0 for 6 pairs, 1 for 3 pairs and 2 for 3 pairs), quantities less than the difference between isolates in the two outbreaks. The second observation emphasizing the distinctness was the presence of many isolates from both the environment and patients collected in 2015, 2016 and 2017 in Tanzania [19] that were also less than 4 SNVs from the 2016 Zambian isolates and 4 or more distant from the2017/2018 Zambian isolates. The 2017/2018 isolates were also clustered with the 2017 isolate from Chiengi. The Chiengi isolate is exceptional (Fig. 3) because it varies from the others by 4 nucleotide variants each of which is unique to the Chiengi isolate. Other isolates with large numbers of unique SNVs occur on long terminal branches (Fig. 3), for example, Mz1 collected in Tanzania in 2016 [20], identified with a box.

**Fig. 4:**
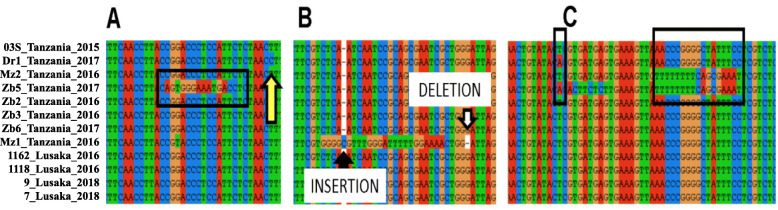
Alignment of selected variable nucleotides from Clustal. Each row is a different isolate. Invariant nucleotides are not shown. The PARSNP vcf file is in Supplemental Table 2. **A**) The arrow points to a single base substitution in isolate DR1, the box surrounds a series of substitutions in Zb5. **B**) The co-occurrence of a single base insertion, a single base deletion and multiple substitutions in Mz1. **C**) The boxes indicate the four possible combinations of two alleles at two loci 15,103 bases apart. The alleles are T & A and TTTTTTTCAGCGAAAT =1, &ACCCGGGGCTATTTCG =2. The genotypes T2, A2, T1 and A1 occurred in 03S, Dr1, Mz2, and Zb5, respectively (Supplemental Table 3)

We examined in detail the SNVs that contributed to the long terminal branch lengths: some occurred as widely spaced single nucleotides, while other appeared in clusters (Fig. 4, Supplementary Tables 3 and 4). The four SNVs in the Chiengi isolate were widely spaced in the genome. A cluster of nucleotides was defined as five or more occurring close together usually with less than ten nucleotides between the variant nucleotides. Eighty-seven clusters were identified with an average of 15 SNVs (range 5 to 38) occurring on average within 36 nucleotides (range 5 to 186). The total number of variable nucleotides in clusters was 43% (1340 of 3143) of all variable nucleotides (Supplemental Table 4). Eighty of the 87 (92%) clusters were found within known genes (Supplemental Table 3). For example, Zb5 (Fig. 4A) has 12 variable nucleotides clustered within 13 nucleotides. Some clusters were flanked by a single base insertion and a single base deletion for example Mz1 (Fig. 4B). Another cluster has 16 variable nucleotides within 27 bases with alleles TTTTTTTCAGCGAAAT called ‘1’ and ACCCGGGGCTATTTCG called ‘2’ (boxed in Fig. 4C) that when examined with a locus with A and T alleles, 15,103 bases away (also boxed in Fig. 4C), was observed to occur in all four allelic combinations, i.e. T1, A1, T2 and A2, in Mz2, Zb5, 03S and Dr1, respectively.

## Discussion

The genetic relatedness of 72 *V. cholerae* isolates collected during 2009/10, 2016 and 2017/18 cholera outbreaks from three districts of Zambia were determined using MLVA and WGS. The three CCs identified by MLVA were temporally distinct from each other (Fig. 1). This demonstrates that the *V. cholerae* strains within a single outbreak were not related to strains from outbreaks in the other years analyzed. These observations are different from findings in several recent studies. In Uganda, three genetically distinct CCS’s were identified among the isolates analyzed and interestingly, multiple CC’s were identified within single outbreaks in time and space [4]. In contrast, a study in Tanzania found that isolates from within a single outbreak were genetically similar [21]. In Cameroon in 2014, two different CCs of *V. cholerae* were responsible for causing two temporally and geographically distinct outbreaks [22].

When our data were analyzed at the ward level within Lusaka, the capital city and an identified cholera hotspot [23], we observed that isolates from the 2016 outbreak came from eleven wards, while the 2017/18 isolates came from three wards (Fig. 2). These three wards (Kanyama, Matero and Raphael Chota) experienced cholera outbreaks during both the 2016 and 2017/18. Clearly, it is feasible that 2016 outbreak could have seeded the next outbreak. However, CCs for these outbreaks were distinct. In the 2016 outbreak, we observed CC2, while in 2017/18, CC3 was seen. Thus, it is less likely that the 2016 isolates seeded the 2017/2018 outbreak. However, cholera affected these same wards in two successive outbreaks, highlighting their vulnerability and the potential for targeted intervention. Additionally, our results identified two serotypes (O1-Inaba from Chiengi and O1-Ogawa from Mpulungu) with related MLVA genotypes. The isolates differed only by a single repeat at two loci (VC0147 and VCA0171). These results are in accord with prior data which indicates that multiple serotypes can be present within a single genetic lineage. However, the distinct serotypes may represent separate transmission events into the two locations [7]. Remarkably, the MLVA genotype found in Mpulungu is the most frequent genotype found in Lusaka in 2017/2018, consistent with the possible spread between the northeastern district and the centrally located capital city.

The WGS analysis revealed that the isolates from 2009 to 2010 (CC1) belonged to T10 and those from 2016 and 2017/18 outbreaks belonged to T13. Isolates from T10 are widespread in East Africa having been found in Uganda, Kenya and Tanzania [4]. T13, the most recent transmission event from south Asia to Africa, now has been confirmed in Zambia after appearing in East Africa (Uganda, Kenya, Somalia, Ethiopia, and Tanzania) and Yemen [22]. Notably, none of the isolates from Zambia belonged to T11 which has been found and has persisted in neighboring Mozambique for at least eight years [24].

At the DNA level, the WGS analysis revealed that variant nucleotides were either widely spaced or clustered (Supplementary Table 3). The widely spaced nucleotide substitutions occurring at dispersed loci are consistent with random mutation. The clustered variant nucleotides are unlikely to be caused by random mutation because five independent mutations are not expected to be clustered within 100 bp, a calculated probability of 10^**− 16**^ (mutation rate per base = 10^**− 6**^, at any of 100 bases = 10^**2**^, multiplied together = 10^**− 4**^, raised to the fourth power for four additional mutations = 10^**− 16**^). The clustered variants are likely to have occurred non-randomly.

The clustered SNVs occurred either as a simple series (Fig. 4A) or co-occurred with other mutations. Remarkably, these SNVs occurred primarily within genes, 92% are in genes. In one case of co-occurrence (Fig. 4B), the clustered variant nucleotides were near a single base insertion and a single base deletion. A single base insertion would shift the reading frame likely leading to a non-functional protein, but a nearby single base deletion would restore the reading frame and potentially preserve the protein function. Small insertion and or deletion events co-occurring with multiple substitutions may be the result of a non-homologous end joining event, a DNA repair mechanism for correcting double strand breaks, as has been seen previously in bacteria [25]. In an alternative case (Fig. 4C), the clustered variants occurred on chromosomes carrying all possible combinations of two alleles at two loci. The presence of all combinations is unlikely to be the result of recurrent mutation (*p* < 10^**− 12**^, for a point mutation) and is more parsimonious with recombination between related isolates [24].

Our results indicate that three separate transmission events were responsible for the introduction of *V. cholerae* into Zambia during this time. The isolates from CC1 were part of the T10 lineage and represent one transmission of *V. cholerae* into Lusaka. The second (CC2) and third (CC3) genetic lineages cluster on distinct branches in the T13 WGS tree. The CC2 isolates cluster with isolates from Tanzania collected in 2016 [20]. The CC3 (2017/2018) isolates cluster with 2015, 2016 and 2017 environmental and patient isolates from Tanzania [19] and the isolate from 2017 collected in Chiengi. The genetic and temporal distinctness of CC1/T10/2010, CC2/T13/2016 and CC3/T13/2017/2018 are consistent with the occurrence of three separate transmission events into Lusaka. The presence of genetically closely related isolates from i) the environment (fish and water) and patients in Tanzania (northeast of Zambia) in 2016 [19], ii) Chiengi located on Lake Mweru in the northeastern Zambia in 2017, and iii) Lusaka in central Zambia in the 2017/2018 outbreak is consistent with the spread of that lineage from Tanzania to Lusaka. The evidence of multiple introductions of cholera into Lusaka and Zambia is not consistent with an endemic strain of cholera that seeds the next outbreak, but rather recurrent spread of *V. cholerae* into Zambia.

There were several limitations to this analysis. One limitation is that finer mapping of *V. cholerae* transmission dynamics cannot be done using WGS because first, there is a lack of diversity (too few SNVs) in our WGS data. We only observed 3 SNVs or fewer in each of the three epidemiological outbreaks which corresponded to an MLVA CC in Lusaka and among a subset of 2015, 2016 and 2017 isolates from Tanzania. Second, we observed up to 2 SNVs differences between separate DNA extractions and sequencing of the same isolate. This makes tracking the transmission of isolates more difficult since more than 4 SNP differences are required to claim that the isolates are distinct. Third, we do not have the GPS coordinates for each isolate nor the exact date of specimen collection to map them in time and space. In the case of Mpulungu and Chiengi in 2017/2018, it would have been beneficial to have more than one isolate from this area with the exact date of collection. Fourth, our study is biased towards isolates from Lusaka since the best equipped laboratories are there. Finally, any additional information and specimens may have improved our ability to define transmission patterns.

## Conclusion

Our genetic analysis of *V. cholerae* isolates revealed three, spatially and temporally distinct genetic lineages in Zambia. We infer that these outbreaks have different origins and must have entered independently into the country from across its borders. Consistent with our observation, on a local level, case-area targeted invention strategies [23, 26, 27] should be employed and at the international and regional level, well-coordinated plans should be put in place to effectively control future cholera outbreaks.

## Supplementary Information

**Additional file 1 Supplemental Figure 1:** Unrooted Phylogram of the Genetic Relatedness of Isolates from Zambia, East Africa and Asia.

**Additional file 2 Supplemental Table 1**: DNA Sequences in This Study.

**Additional file 3 Supplemental Table 2**: Number of Single Nucleotide Variants Between Pairs of Isolates.

**Additional file 4 Supplemental Table 3**: Single Nucleotide Variants Found in Each Isolate and the Gene in Which the Single Nucleotide Variants Occurred.

**Additional file 5 Supplemental Table 4**: List of Clusters of Single Nucleotide Variants in Identified Supplemental Table 3

## Data Availability

DNA sequence data are available in Genbankhttps://**www.ncbi.nlm.nih.gov/**, accession number PRJNA701309.Most data generated or analysed during this study are included in this article and its supplementary information files. DNA, scripts and any data not in public databases or the articles used and/or analysed during the current study are available from the corresponding author on reasonable request.
