# Supplementary material for: Three transmission events of Vibrio cholerae O1 into Lusaka, Zambia
Source: BMC Infect Dis. 2021 Jun 14;21:570. doi: 10.1186/s12879-021-06259-5 (PMC8200794; doi:10.1186/s12879-021-06259-5)
Supplement: Supplementary file 1 — Additional file 1 Supplemental Figure 1: Unrooted Phylogram of the Genetic Relatedness of Isolates from Zambia, East Africa and Asia. [file 12879_2021_6259_MOESM1_ESM.docx]

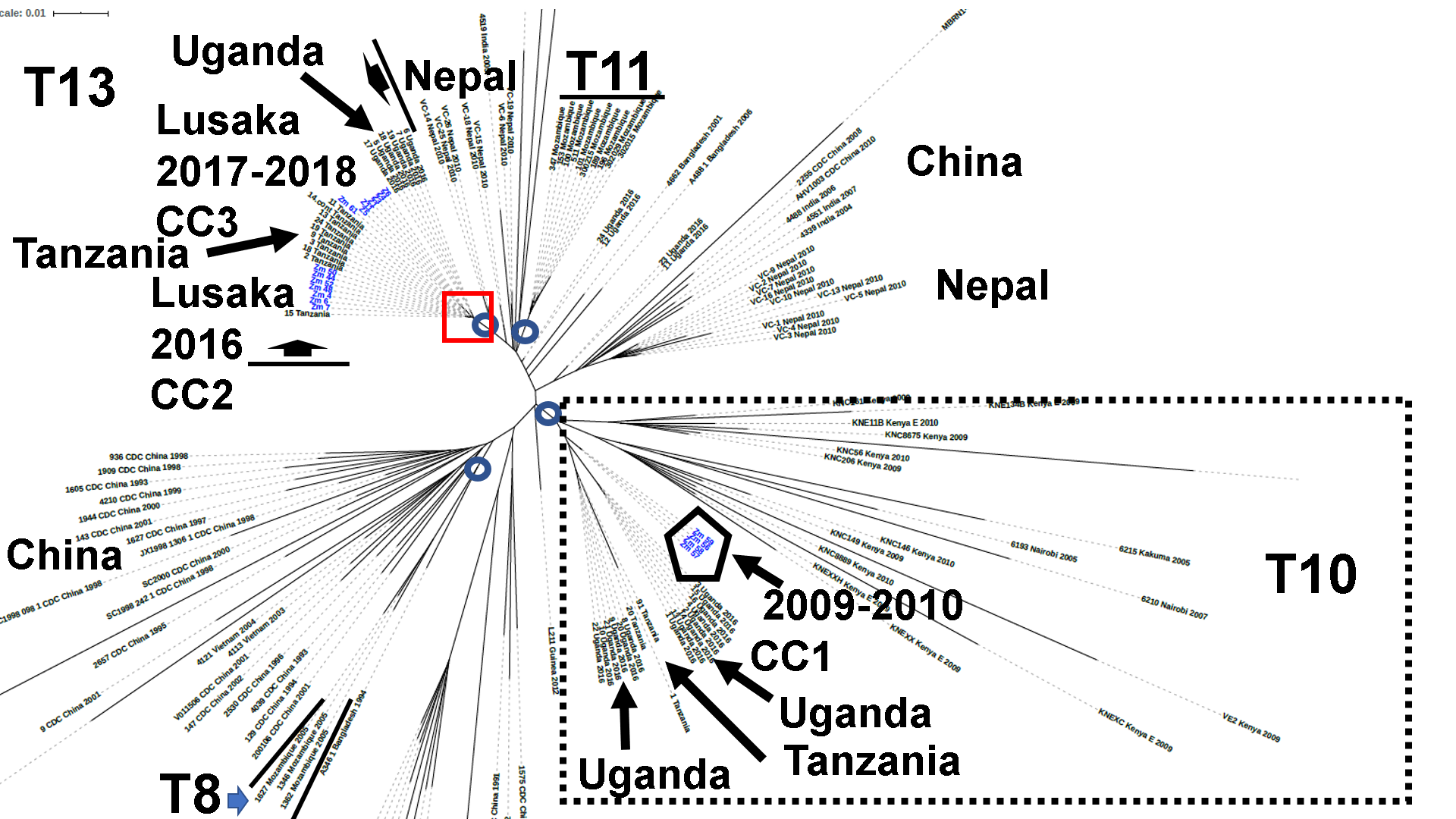


**Supplemental Figure 1:** **Unrooted Phylogram of the Genetic Relatedness of Isolates from Zambia, East Africa and Asia.** The solid lines are proportional to the number of SNVs between nodes. The dotted lines connect the isolate name (Zambian isolates are blue) to the tip of the phylogram. The blue circles indicate the branches leading to transmission events (eg T10, T13) from south Asia to east Africa. Inside the black dotted rectangle are the isolates from T10. These isolates have extensive genetic diversity as seen by the long lines. In contrast, in the pentagon the isolates from Zambia collected in the 2009 and 2010 outbreak have minimal genetic diversity consistent with a founder effect. Inside the red solid square are the isolates from T13. These isolates have minimal genetic diversity, as well. An expanded version of this region can be found in Figure 3.
